# Climate change and vertically transmissible infections in maternal and neonatal populations: a systematic review

**DOI:** 10.3389/fgwh.2026.1931882

**Published:** 2026-09-23

**Authors:** Kimberly Sarah Mc Alpine, Tim Groteclaes, Danielle Travill, Margaret Brennan, Natasha Lalloo, Ruvimbo Forget, Carla Devantier-Du Plessis, Lebohang Radebe, Nicholas Brian Brink, Ashraf Coovadia, Matthew Francis Chersich, Darshnika Pemi Lakhoo

**Affiliations:** 1 Wits Planetary Health Research, Faculty of Health Sciences, University of Witwatersrand, Johannesburg, South Africa; 2 Department of Paediatrics and Child Health, School of Clinical Medicine, Faculty of Health Sciences, University of the Witwatersrand, Johannesburg, South Africa; 3 Center for Pediatric and Adolescent Medicine, Department of Neonatology, University Hospital Heidelberg, Heidelberg, Germany; 4 Heidelberg Institute of Global Health (HIGH), Faculty of Medicine and University Hospital, Heidelberg University, Heidelberg, Germany; 5 Department of Public Health and Primary Care, Trinity College Dublin, Dublin, Ireland

**Keywords:** ambient temperature, climate change, drought, group B streptococcus, HIV, mother-to-child transmission, neonatal infections, pregnant women

## Abstract

**Background:**

Climate change influences infectious disease dynamics and disrupts maternal and neonatal health services, both of which are pathways through which the risk of vertically transmissible infections may increase.

**Methods:**

We systematically reviewed evidence on the association between climate-related environmental exposures and infection-related outcomes relevant to vertical transmission in pregnant or intrapartum women, neonates, and infants. Screening, extraction, and risk-of-bias assessment were performed independently in duplicate. Owing to substantial heterogeneity, findings were synthesised narratively. GRADE scoring was used to assess certainty of evidence.

**Results:**

Of 4,579 records identified, 42 full texts were reviewed and 6 studies included. Studies examined drought/famine, temperature, humidity, and climate zone in relation to HIV, group B streptococcus (GBS), varicella-zoster virus (VZV), and bacterial meningitis. No study reported vertical transmission as an outcome. Higher temperature and humidity were associated with increased maternal GBS colonisation. Three studies suggested cooler and/or drier conditions were associated with increased VZV transmission or earlier serological acquisition. One study found drought-associated famine was linked to increased HIV prevalence in pregnant women in rural Malawi. Certainty of evidence was very low across outcome groupings.

**Conclusions:**

This review found evidence suggesting climate-related exposures may be associated with infections relevant to vertical transmission through pathogen-specific biological pathways, broader social disruption, and deprivation. There is currently insufficient evidence to determine the impact of climate change on vertical transmission or related outcomes. Closing this gap should be a priority for research and climate adaptation in maternal and neonatal health.

**Systematic Review Registration:**

https://www.crd.york.ac.uk/PROSPERO/view/CRD420251124435, PROSPERO CRD420251124435.

## 1 Introduction

Climate Change is considered one of the largest global health challenges of the 21st century (1, 2). Global warming trends are predicted to exceed 1.5 °C within the next decade (3). Global warming is accompanied by increasing frequency and severity of extreme weather events (EWEs) including heatwaves, droughts, wildfires, and floods (1–4). Climate change alters pathways that affect infectious disease dynamics, strains healthcare infrastructure and disrupts health service delivery (1–10), with serious implications for populations facing increased biological and social vulnerability (4, 11). These disruptions include disruptions to antenatal care (ANC) and delayed diagnosis and treatment of infections, potentially worsening maternal and neonatal health (MNH) outcomes (12, 13).

Pregnant women and neonates are especially vulnerable to increasing temperatures and heat extremes associated with climate change, particularly those in low- and middle-income settings where health systems are fragile and climate exposure is high (12–15). Pregnancy is associated with physiological and immunological adaptations that may heighten susceptibility to heat (16, 17), while neonates have immature immune systems and limited thermoregulatory capacity (18, 19). These biological vulnerabilities are compounded in sub-Saharan Africa and South Asia, where the burden of climate-sensitive disease is highest, access to protective health services is most limited and adaptive capacity is low.

Vertical transmission refers to the transmission of infectious disease from one generation to another either in-utero, intrapartum or postpartum via breastfeeding (20). Vertically transmitted infections (VTIs) remain important contributors to maternal and neonatal morbidity and mortality globally. These include HIV and other sexually transmitted infections (e.g., syphilis, chlamydia, gonorrhoea, trichomoniasis, and HPV), TORCH pathogens [Toxoplasmosis, Rubella, Cytomegalovirus (CMV), and Herpes Simplex Virus (HSV)], hepatitis B virus, and group B streptococcus (GBS) (21). In 2021, HIV and syphilis had age-standardized prevalence rates of 830.87 and 1,057.32 per 100,000 among women of childbearing age respectively, with over 95% of paediatric HIV infections attributable to mother-to-child transmission (22). New HIV infections among infants and children have declined since 2010, however in recent years progress in reducing these new infections has stalled (23). Untreated or inadequately treated syphilis in pregnancy results in adverse birth outcomes in 50%–80% of affected pregnancies (24). Additionally, GBS is the leading cause of neonatal sepsis worldwide (25, 26). The burden is disproportionately concentrated in low- and middle-income settings where health systems are fragile, and access to screening, prevention, and treatment is limited (27).

Climate change influences infection risk, transmission, and disease severity through multiple pathways (2, 4–8, 13, 14, 28). At the biological level, heat and EWEs may directly influence pathogen survival and proliferation, while also increasing host susceptibility through mechanisms such as skin barrier disruption and impaired immune function. At the behavioural and socioeconomic level, food insecurity and population displacement driven by EWEs can push vulnerable populations into higher-risk situations, including transactional sex and disrupted social networks, that facilitate transmission. A third pathway operates through the disruption of healthcare access and service delivery, whereby EWEs may disrupt water and sanitation infrastructure, increase pathogen exposure, and strain healthcare infrastructure, with potential implications for MNH programmes including antenatal care, screening, and prevention of mother-to-child transmission services. Despite growing interest in the relationship between climate change and infectious diseases, evidence on climate change and VTIs has not yet been systematically collated. This gap was underscored by an overview of reviews that identified climate-sensitive infectious diseases and MNH as an area without existing evidence synthesis (15). The climate–HIV relationship has been partially explored (29–31), but without attention to maternal HIV prevalence, vertical transmission, or neonatal outcomes. Evidence for other VTIs remains dispersed across pathogens, geographical regions, and exposures, limiting the understanding of their association with climate change.

Examining the relationships between climate change and VTIs is important for strengthening MNH systems in a warming world. This systematic review aimed to identify, describe, and synthesise the available evidence on associations between climate change, specifically heat and EWEs, and VTIs in maternal and neonatal populations. Secondary objectives were to compare findings across pathogen groups, exposure types, and geographical settings, and to identify evidence gaps to inform future research and climate adaptation planning.

## 2 Methods

### 2.1 Study design and reporting standards

The review adhered to the Preferred Reporting Items for Systematic Reviews and Meta-Analyses (PRISMA) 2020 statement (32). The review protocol was prospectively registered with PROSPERO (CRD420251124435).

### 2.2 Eligibility criteria

Eligibility was defined using a Population–Exposure–Outcome–Study design (PEOS) framework.

#### 2.2.1 Population

We included studies involving pregnant or intrapartum women, neonates (≤28 days), or infants up to one year of age. Studies restricted to non-pregnant populations, males, non-human subjects, or children older than one year were excluded.

#### 2.2.2 Exposure

Eligible studies assessed climate-related environmental exposures, including ambient heat metrics and EWEs such as heatwaves, droughts, floods, or wildfires. Eligible exposures included both formally defined climate change indicators and ambient meteorological conditions representing climate sensitivity, that is, associations between current weather patterns and outcomes that are relevant to understanding the potential health impacts of climate change. Studies examining indoor or occupational heat exposure, air pollution alone, or vector indices, such as vector abundance and infection rates, not directly linked to a climate metric were excluded.

#### 2.2.3 Outcomes

Included outcomes were infection-related endpoints relevant to vertical transmission. Studies reporting general pregnancy or birth outcomes without a specific infection endpoint were excluded.

#### 2.2.4 Study design

Observational designs (cohort, case–control, cross-sectional, ecological, and time-series studies) and interventional studies were eligible. Laboratory or animal studies, purely modelling studies, case reports or series with fewer than ten cases, reviews, editorials, and commentaries were excluded.

### 2.3 Databases and search strategy

Electronic searches were conducted in English in PubMed (via MEDLINE), Web of Science Core Collection, and Scopus from database inception to 14 October 2025. The search combined controlled vocabulary terms and free-text terms across three domains, namely: (i) climate change and EWEs, (ii) maternal, neonatal, or infant populations, and (iii) VTIs, including HIV, sexually transmitted infections, TORCH pathogens, hepatitis B virus, and GBS. Full search strategies are provided in the Supplementary Material. Citation searching of included records was performed to identify additional eligible references.

### 2.4 Study selection

Records retrieved from database searches were imported into Covidence systematic review software for deduplication and screening. Duplicates were identified using Covidence's automated algorithm and verified manually. Title, abstract and full-text screening was conducted independently by two reviewers, with discrepancies resolved through discussion between reviewers, or by a third reviewer when agreement could not be reached.

### 2.5 Data extraction

Standardised data extraction was performed independently in duplicate using a structured form within Covidence. Disagreements were resolved through discussion or third-reviewer adjudication. Extracted items included: study identifiers (authors, year, journal, and funding source); study design and setting (country, design, recruitment period, geographical region, and climate zone); population characteristics (eligibility criteria, and sample size); exposure characteristics (exposure type, definition, metric, window, and data source); outcome domain, definition and measurement; effect estimates and precision measures; and information required for risk-of-bias assessment. No statistical imputation was undertaken for missing data.

### 2.6 Data synthesis

Synthesis methods followed the PRISMA 2020 statement (32), and the Synthesis Without Meta-analysis (SWiM) reporting guideline (33). Eligibility of studies for each synthesis group was determined by tabulating pathogen category, exposure type, population, and outcome domain against the planned groupings pre-specified in the protocol.

Given substantial heterogeneity across included studies in design, exposure metrics, exposure windows, pathogens, outcome definitions, and effect measures, meta-analysis was not considered appropriate and was not undertaken. All findings were synthesised narratively, using direction of effect as the primary synthesis metric, categorised as increased risk, decreased risk, or no clear association. As no included study reported vertical transmission as a direct outcome, findings are described as infection outcomes relevant to vertical transmission throughout the synthesis.

### 2.7 Risk-of-bias assessment

Risk of bias was assessed independently in duplicate. The JBI Critical Appraisal Checklist for Cohort Studies was applied for cohort studies, cross-sectional studies and prevalence studies (34). Items pertaining to follow-up (items 8–10) were rated as “not applicable” for non-cohort studies. We considered this approach as valid as the substantive appraisal domains addressed by the JBI cross-sectional (35) and prevalence (36) checklists are covered within the cohort checklist. Each JBI item was rated “yes”, “no”, “unclear”, or “not applicable” and then interpreted as having low, moderate, high, or not applicable risk of bias accordingly.

For retrospective time-series studies, the JBI Cohort Checklist was not considered sufficient, as it does not address design-specific risks of bias inherent in time-series regression analyses. These studies were therefore appraised using structured quality criteria for time-series regression studies, operationalised over nine-domains covering study base, exposure measurement, outcome measurement, control for season and long-term trend, control for other time-varying confounders, lag specification, model fit, and precision reporting (37). Each domain was rated as “adequately addressed”, “partially addressed”, “not addressed”, or “not applicable”, which was then interpreted as having low, moderate, high, or not applicable risk of bias accordingly.

Risk of bias assessments were visualised using “traffic light” plots created using robvis (38).

### 2.8 Certainty of evidence

The certainty of evidence for each pathogen–exposure–outcome grouping was assessed using the GRADE approach adapted for narrative synthesis. Assessments considered five domains: study limitations (risk of bias), inconsistency, indirectness, imprecision, and potential publication bias (39).

## 3 Results

### 3.1 Study selection

Database searches identified 4,576 records, to which citation searching contributed a further 3 references, giving a total of 4,579 records. Following removal of 1,546 duplicates, 3,033 records were screened at title and abstract, of which 2,991 were excluded. Full texts for the remaining 42 records were successfully retrieved and assessed for eligibility. Thirty-six were excluded due to ineligible study design (15), outcomes (9), patient population (8), and exposures (4) (Supplementary Table 1). Six records met all eligibility criteria and were included in the review (Figure 1).

**Figure 1:**
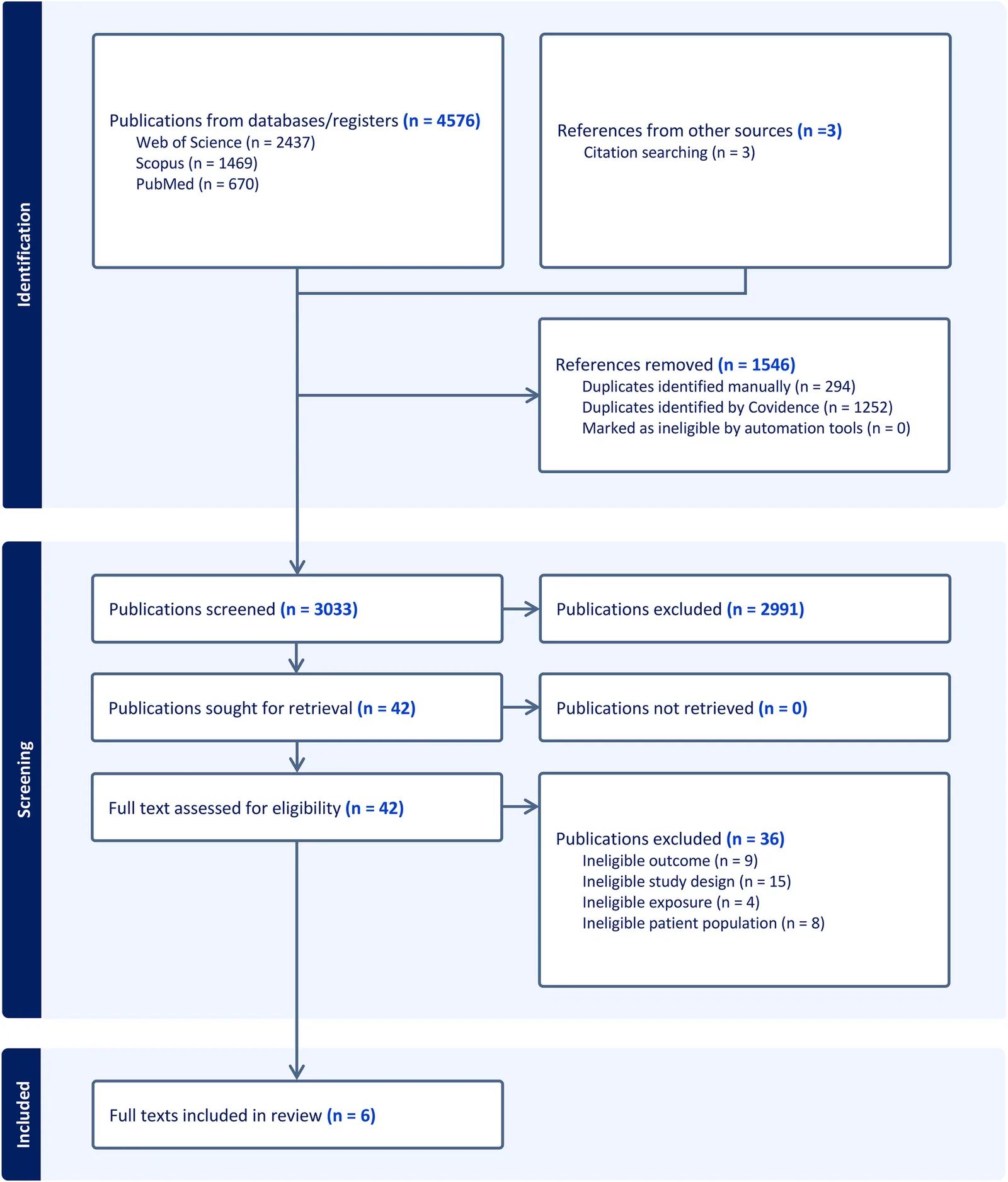
PRISMA flow diagram of the record selection process.

### 3.2 Characteristics of included studies

The six included records were published between 1994 and 2015, and were conducted in Eswatini (40), Greece (41) Hong Kong (40), Malawi (42), Spain (43), and Thailand (44), spanning low-(*n* = 1) (42), lower-middle- (*n* = 2) (40, 44), upper-middle- (*n* = 1) (41), and high-income countries (*n* = 2) (43, 45), across four World Health Organisation (WHO) regions (Table 1). Four studies were conducted in temperate climate zones (41, 43, 45), one in a tropical climate zone (44), and two spanned multiple zones (40, 42). Three records used ecological level exposure assignment (40, 42, 44). One record used GIS methods to assign individual residential exposure to the nearest meteorological station (43). Two time-series records linked aggregate hospitalisation counts to meteorological time-series data (41, 45). Included populations comprised pregnant women (*n* = 2) (42, 43), and broader paediatric cohorts encompassing infants (*n* = 1) (44), or infants and neonates (*n* = 3) (40, 41, 45).

**Table 1:** Characteristics of included records.

Characteristic	Categories	N
Continent	Africa	2
	Europe	2
	Asia	2
Köppen climate zone	A: tropical	1
	B: arid	0
	C: temperate	3
	D: continental	0
	E: polar	0
	Multiple^a^	2
Income level	Low income	1
	Low-middle income	2
	Upper-middle income	1
	High income	2
Population group^b^	Pregnant Women	2
	Neonate	3
	Infant	4
Year of Publication	1990–1999	1
	2000–2009	1
	2010–2019	4

a The Malawi study covered tropical and temperate zones (42), while the Eswatini study was dominated by dry and temperate zones (40).

b Three records included neonates and infants (40, 41, 45), hence population group totals sum to 9, not 6.

Findings are presented as a narrative synthesis organised by pathogen group and climate exposure (Table 2).

**Table 2:** Summary of findings table.

Main Author (Year)	Study setting	Population	Disease	Climate exposure	Outcome measurement	Primary finding
Loevinsohn (2015) (42)	Malawi	Pregnant women	HIV	Famine/Drought	Prevalence	Significant increase in HIV prevalence at rural sites, non-linearly associated with the extent of drought-associated hunger in the district. Prevalence decreased at semi-urban and urban sites.
Ford et al. (1994) (40)	Eswatini (Swaziland)	Infants and neonates	Bacterial meningitis	Drought	Incidence	A significant increase in incidence coinciding with a period of severe drought in the country.
Dadvand et al. (2011) (43)	Barcelona, Spain	Pregnant women	GBS	Daily temperature, relative humidity, & heat index	Prevalence	Increased prevalence of GBS colonisation at higher ambient temperatures and relative humidity.
Critselis et al. (2012) (41)	Athens, Greece	Paediatric patients (included infants)	VZV	Air temperature ( °C), relative humidity (%), and wind speed (m/s)	Incidence	Incidence of hospitalized VZV cases was inversely associated with temperature and positively associated with wind speed.
Chan (2011) (45)	Hong Kong, China	Children and adolescents aged <18 yrs (including infants)	VZV	Monthly mean relative humidity, monthly mean air temperature, and monthly total rainfall	Incidence	Monthly mean relative humidity was significantly and inversely associated with hospitalisation (*β*=−2.67, *p* = 0.002), meaning cases increased as humidity decreased; this effect was most pronounced during the cool season.
Lolekha et al. (2001) (44)	Thailand	People aged 9 mo. to 29 yr (including infants)	VZV	Regional climatic variation (mean/ maximum/ minimum temperature, annual rainfall, & mean humidity)	Prevalence	Seroprevalence was significantly higher in cooler, high-altitude regions (Northern region: 33.3% among children aged 9mo–4 yr) compared to warmer tropical regions (Southern region: 6.2% for the same age group), suggesting higher temperatures and humidity may be associated with reduced viral transmission.

HIV, human immunodeficiency virus; VZV, Varicella Zoster Virus; GBS, group B streptococcus; mo., month; yr, year.

### 3.3 Risk of bias

Among the JBI-appraised studies (Figure 2), Dadvand et al. (43) was judged low risk of bias and was the most methodologically robust study, with individual-level covariate adjustment, validated microbiological outcome measurement, and appropriate spatial exposure linkage. However, the exposure window was selected empirically as the period yielding the lowest Akaike Information Criterion (AIC), introducing some risk of overfitting. Lolekha et al. (44) and Loevinsohn (42) were judged moderate risk of bias. Both used appropriate outcome assessment and analytical approaches, but causal inference was limited by ecological exposure assignment and design-level constraints. For Lolekha et al. (44), additional concerns related to convenience-based recruitment and potential conflict of interest, while Loevinsohn (42) was limited by the complexity of drought-famine exposure pathways and district-level exposure classification. Ford et al. (40) was judged high risk of bias due to the absence of a formal climate exposure measure, lack of confounder adjustment, and insufficient statistical rigour.

**Figure 2:**
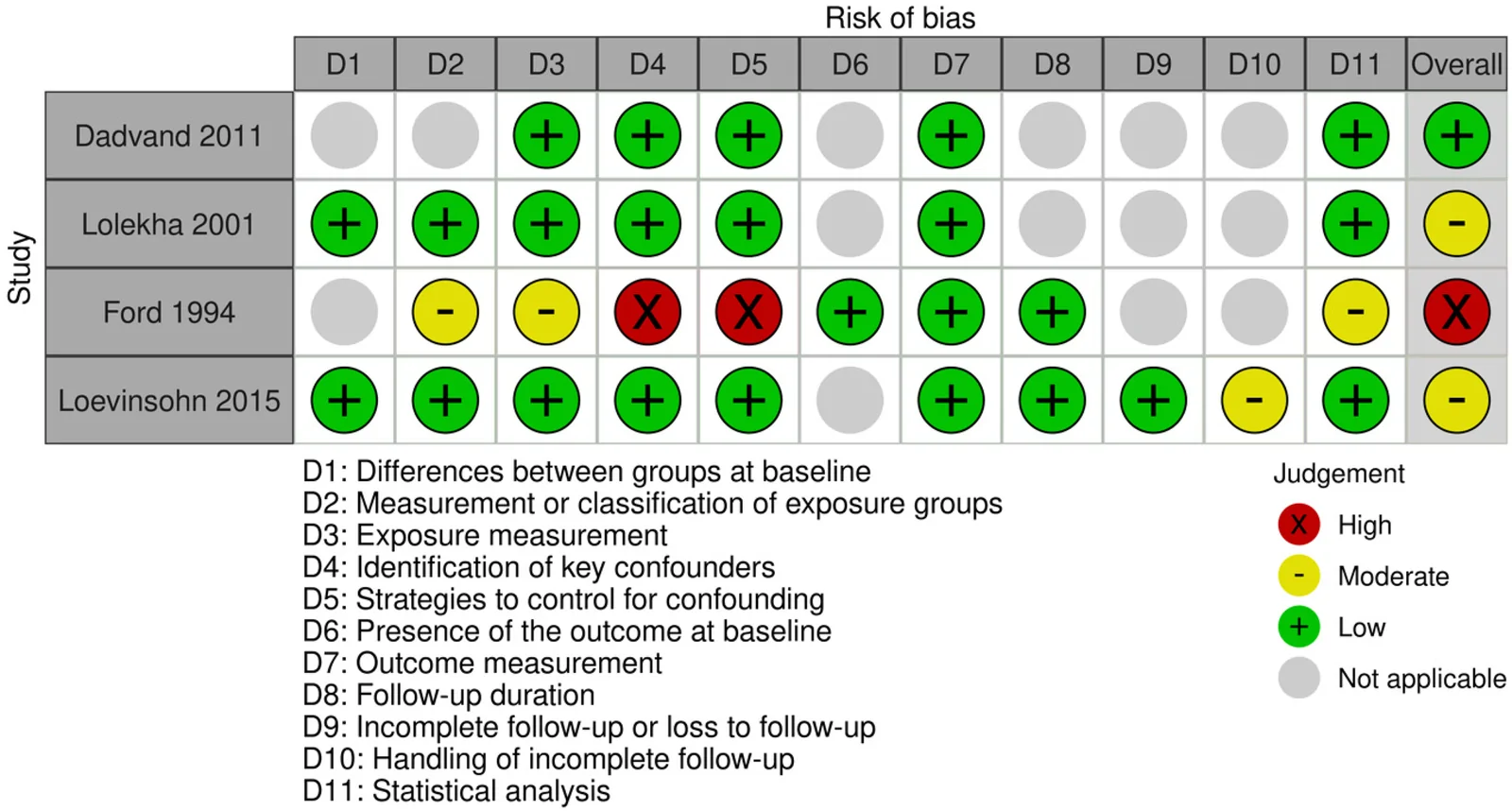
Risk of bias assessment across five studies using the JBI cohort checklist.

Both time-series studies, Chan et al. (45) and Critselis et al. (41), were judged high risk of bias, primarily because of inadequate control for time-varying confounding (Figure 3). Chan et al. (45) used stronger statistical modelling, including Poisson GEE with autoregressive terms, but remained limited by use of a single tertiary-hospital dataset, ICD-9-based case identification, restriction to hospitalised cases, and limited adjustment for non-climatic confounders. Critselis et al. (41) had broader methodological limitations, including incomplete reporting of model parameters, limited model checking, lack of confidence intervals for key estimates, and insufficient description of exposure measurement.

**Figure 3:**
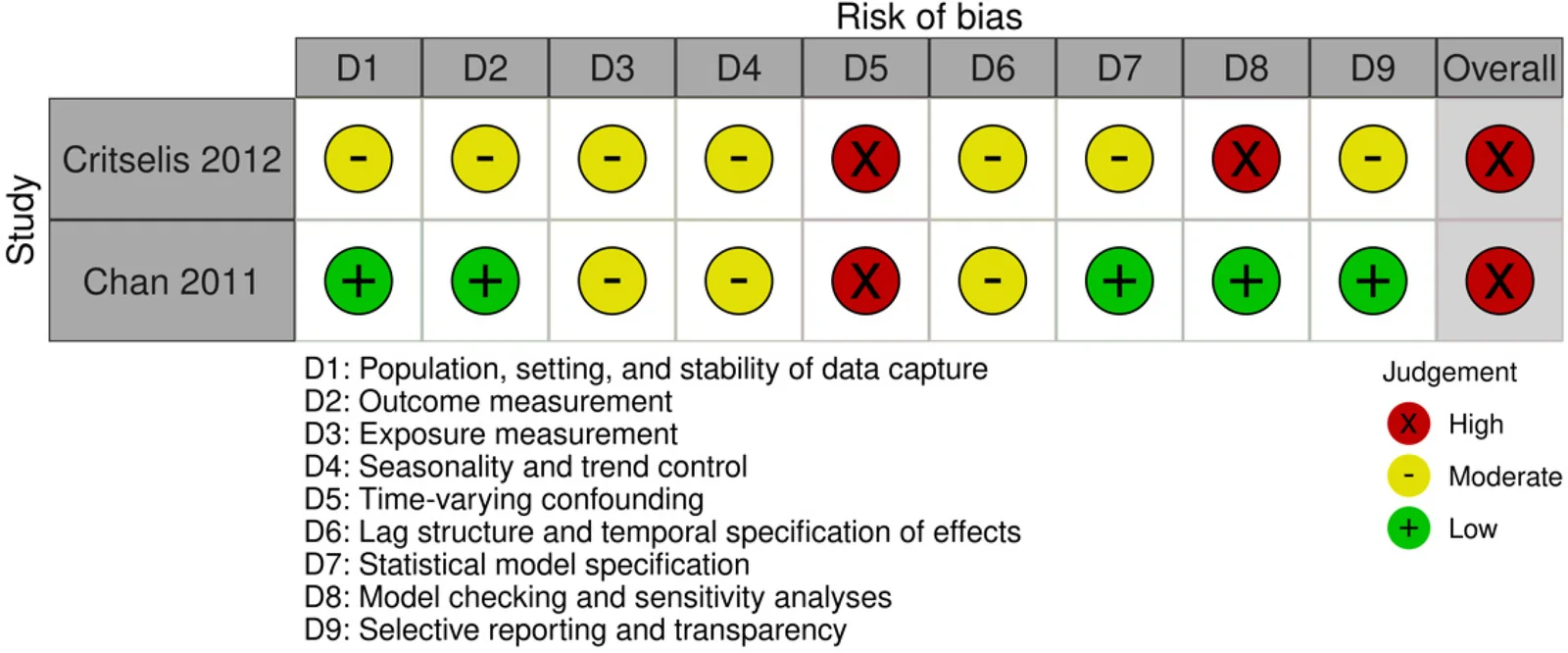
Risk of bias assessments for the two time series regression studies.

Overall, the main sources of bias were ecological or poorly specified exposure measurement, limited confounder control, and constraints inherent to descriptive and retrospective designs. These limitations reduce confidence in the observed climate–infection associations. No studies were excluded from the narrative synthesis on the basis of risk of bias scoring, as the small number of eligible studies meant that exclusion would have precluded any meaningful synthesis. Instead, methodological limitations were taken into account in the interpretation of findings and the GRADE certainty ratings assigned to each outcome grouping.

### 3.4 Drought, famine, and infection outcomes in maternal and neonatal populations

Two studies examined infection outcomes in relation to drought or drought-associated famine conditions, one focusing on HIV prevalence in pregnant women and one on bacterial meningitis incidence in neonates and infants (Figure 4).

**Figure 4:**
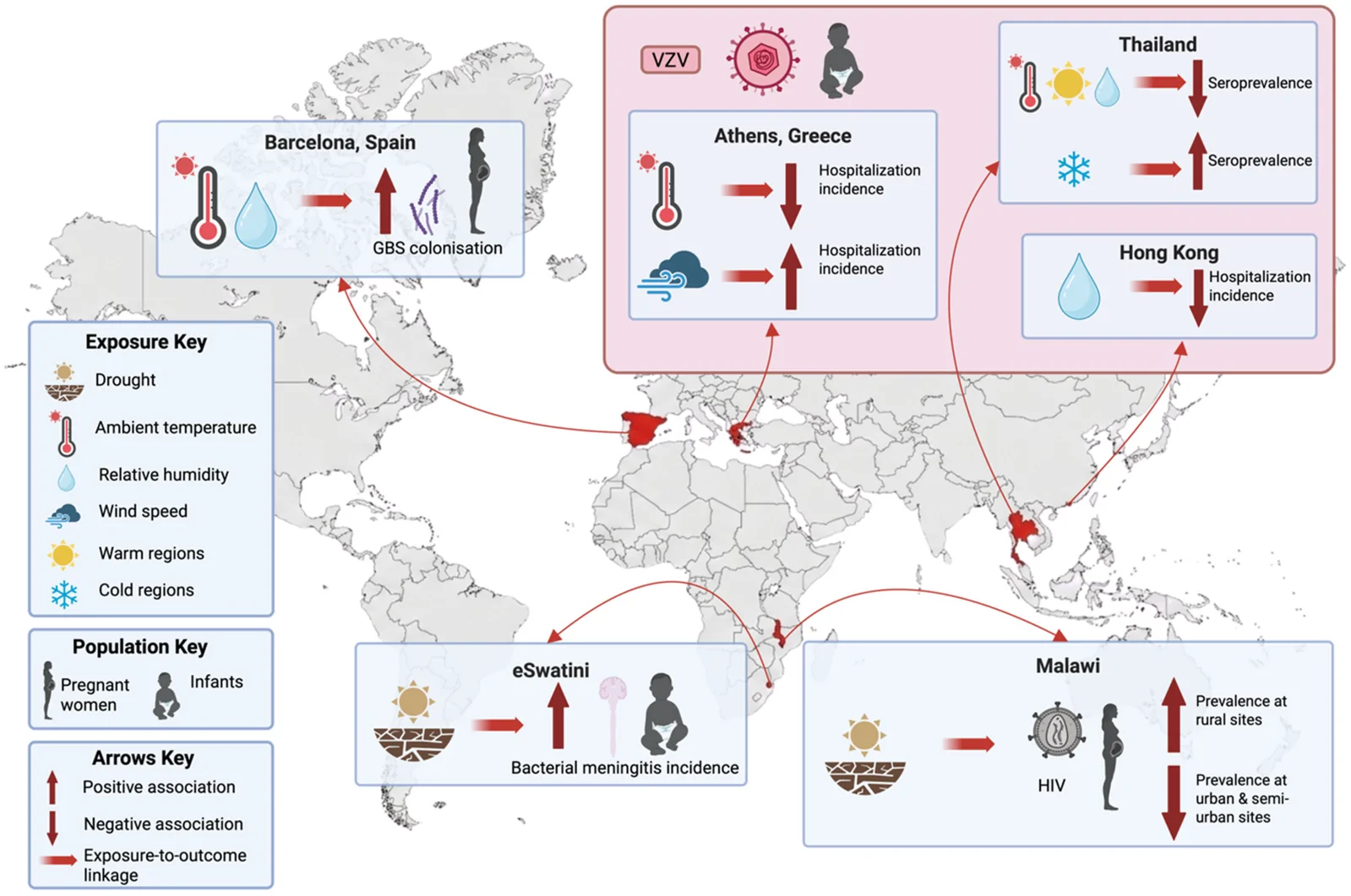
Geographic distribution and direction of associations between climate exposures and vertically transmissible infection outcomes across included studies. Each study is mapped to its country of origin with arrows indicating the direction of the association observed between climate exposure and infection outcome. The pink-shaded region groups the studies reporting VZV outcomes. Barcelona (43): higher ambient temperature and relative humidity were positively associated with maternal GBS colonisation. Athens (41): ambient temperature was inversely associated with VZV hospitalisation incidence; wind speed showed a positive association. Thailand (44): VZV seroprevalence was lower in warmer regions and higher in cooler regions. Hong Kong (45): relative humidity was inversely associated with VZV hospitalisation incidence. Eswatini (40): bacterial meningitis incidence increased during a period of severe drought. Malawi (42): drought-driven famine was associated with increased HIV prevalence at rural antenatal sites and decreased prevalence at urban and semi-urban sites. Created in BioRender. Mc Alpine, K. (2026) https://BioRender.com/xu8jflh.

Loevinsohn (42) used the 2001–2003 Malawi famine, driven by drought, poor harvests, and policy failures, as a natural experiment, evaluating HIV prevalence in pregnant women through anonymous serological testing of first-visit antenatal attendees. District-level exposure was operationalised as the modelled proportion of rural households requiring food assistance during the peak famine period.

Adjusted multilevel models identified a 20.2% increase in HIV prevalence at rural sites between pre-famine and famine/post-famine periods (*P* = 0.016), with a non-linear relationship to hunger severity: little change was observed in areas in which less than 25% of households required food assistance, but HIV prevalence increased markedly above this threshold. Semi-urban and urban sites showed contrasting declines of 21.7% and 14.8% respectively (*P* = 0.006), consistent with the authors' proposed mechanism of drought-driven rural-to-urban migration of young farming women, whose lower baseline HIV prevalence relative to urban populations produced a statistical dilution effect at non-rural sites, creating a compositional shift rather than a true decline in prevalence. However, these migrant women faced a nearly doubled individual infection risk.

Ford et al. (40) reported an 18-month prospective study of bacterial meningitis across four regional hospitals in Eswatini (February 1991–July 1992). Of 85 included cases, 41 were infants and 6 were neonates. A significant increase in incidence was identified during May–July 1992 during a severe drought, compared with the same period in 1991 (32 versus 12 cases; *χ*² = 4.98, *P* = 0.026). Drought exposure was neither formally defined nor quantified, no formal exposure–outcome analysis was performed, and causative organisms were not specified by drought period or age group.

### 3.5 Ambient temperature, humidity, wind speed and infection outcomes

Four studies examined the association between ambient meteorological conditions and infection outcomes in pregnant women and paediatric populations including infants and neonates. This group contains evidence on two biologically distinct pathogens, GBS and varicella-zoster virus (VZV) (Figure 4).

#### 3.5.1 Group B streptococcus colonisation and gestational climate

Dadvand et al. (43) investigated associations between ambient temperature, relative humidity, wind speed, and maternal GBS colonisation in 7,976 pregnant women with full-term singleton deliveries in Barcelona (2001–2005). Colonisation was determined by routine vaginorectal swabbing at 36 ± 1 weeks. Multivariable logistic regression identified significant associations with temperature (OR 1.02 per 1 °C, 95% CI = 1.01–1.03; *P* = 0.012), relative humidity (OR 1.03 per 1% increase, 95% CI = 1.01–1.05; *P* = 0.001), and heat index (OR 1.01 per unit, 95% CI = 1.01–1.02; *p* = 0.009), each averaged across gestational weeks 32–36. Colonisation risk was 21% higher at the end of the hottest versus coolest month and 34% higher with elevated humidity. Categorical analysis suggested threshold effects above 15.4 °C, 72.7% relative humidity, and a heat index of 14.6 °C, though the dose–response relationship did not increase monotonically across quartiles. A sensitivity analysis found no significant effect of seasonality (ANOVA *P* = 0.098), suggesting the association was not explained by season alone.

#### 3.5.2 Varicella-zoster virus transmission and meteorological conditions

Chan et al. (45) in Hong Kong, Critselis et al. (41) in Athens, and Lolekha et al. (44) in Thailand examined the relationship between meteorological variables and VZV epidemiology in paediatric populations that included infants and neonates. Across all three, cooler and less humid conditions were consistently associated with higher VZV transmission or earlier serological acquisition.

Chan et al. (45) conducted a retrospective time-series analysis of 598 varicella hospitalisations in children under 18 years at a tertiary paediatric referral centre in Hong Kong (2004–2008), of whom 64 (10.7%) were aged six months or under. Using Poisson regression with Generalized Estimating Equation (GEE) and autoregressive modelling, mean relative humidity within the same month (lag 0) was a significant inverse predictor of monthly hospitalisation counts across the full cohort (*β* = −2.67, 95% CI = −4.34 to −1.01; *P* = 0.002), with the association strongest in the cool season (October–March: *β* = −3.02, 95% CI = −5.47 to −0.58; *P* = 0.015) but non-significant in the hot season. Rainfall at lag 0 was also inversely correlated with case counts (Spearman r = −0.294; *P* < 0.05). Temperature showed no significant independent association. No age-group specific estimates were reported.

Critselis et al. (41) performed a 22-year retrospective time-series analysis (1982–2003) of 2,366 paediatric varicella hospitalisations at a tertiary children's hospital in Athens, including neonates and infants under one year. Generalised linear modelling identified a significant inverse association between mean monthly air temperature and hospitalisation frequency (*β* = −0.030, SE = 0.008; *P* < 0.001), though not significant after adjusting for seasonality and secular trend. Relative humidity showed no independent association (*β* = −0.001; *P* = 0.916), while wind speed was positively and independently associated with hospitalisation rates (*β* = 0.181, SE = 0.070; *P* = 0.009), with each 1 m/s increase associated with a 20% higher probability of hospitalisation.

Lolekha et al. (44) examined VZV seroprevalence across four climatically distinct regions of Thailand in a cross-sectional study of 2,093 participants aged 9 months to 29 years. Among the youngest age group (9 months–4 years), seroprevalence was markedly lower in the warmer Southern (6.2%) and Central (11.3%) regions than in the cooler Northern region (33.3%), with the Northeast intermediate (12.0%). No formal between-region comparisons were reported for this subgroup. The authors suggested that lower seroprevalence in warmer regions was potentially due to inhibitory effects of high temperature and humidity on viral transmission.

### 3.6 Certainty of the evidence

Certainty of the evidence was assessed using GRADE for three analytic pathogen–exposure groupings (Supplementary Table 2) (39). Ford et al. (40) was excluded from formal GRADE assessment due to aetiological uncertainty, undefined climate exposure, and no quantified exposure–outcome estimate.

Certainty was rated very low across all assessed groupings. For VZV-related outcomes, downgrading was driven by risk of bias, indirectness, and imprecision, particularly because no study directly assessed congenital infection or vertical transmission, and outcome reporting was not specific to maternal or infant populations. For maternal GBS colonisation and for drought/famine and HIV prevalence, downgrading was driven primarily by indirectness, as both outcomes represent maternal proxies rather than confirmed neonatal infection or vertical transmission. Additionally, these groupings were downgraded for suspected publication bias because only a single study contributed evidence.

## 4 Discussion

This systematic review identified six studies examining associations between climate-related exposures and infection outcomes relevant to vertical transmission in pregnant women, neonates, and infants. Across three outcome groupings, the direction of findings was internally coherent. Drought-driven famine was associated with increased HIV prevalence in pregnant women in rural Malawi; higher ambient temperature and humidity were associated with increased maternal GBS colonisation during pregnancy; and lower ambient temperature and humidity were associated with increased VZV infection burden in paediatric populations including infants. These associations suggest that climate plausibly influences the maternal–neonatal infectious disease interface through multiple pathways, even if the magnitude and mechanisms differ across pathogens. The overall certainty of evidence was very low across all groupings, possibly reflecting the early and fragmented state of the literature, rather than absence of effect. One study contributed contextual but not analytic evidence due to unresolved eligibility regarding causative organisms and undefined climate exposure.

The most consequential finding of this review is not any single association but the considerable gap in the literature. No included study was designed to examine climate change attribution, and none measured vertical transmission as a direct outcome. More broadly, no study has yet examined whether climate-related exposures modify the risk of vertical transmission of any pathogen, and none reported confirmed congenital infection or neonatal infection-attributable mortality as an outcome. These absences define the boundaries of what can currently be concluded, and closing these gaps should be a priority for both research and climate adaptation in maternal and neonatal health.

Loevinsohn's (42) association between drought-driven famine and increased HIV prevalence among pregnant women attending rural antenatal clinics in Malawi operated through a cascade of socioeconomic and behavioural disruptions mediated by food insecurity. The proposed causal pathway includes drought-related crop failure, resource scarcity, and food price shocks that forces vulnerable rural women into “situations of risk”, such as transactional sex, early marriage to older higher-risk partners, and migration. This pathway is supported by evidence linking food insecurity to increased sexual risk behaviour and reduced negotiating power around safe sex (29, 31). The natural experiment design affords stronger causal leverage than conventional observational studies (46), and the corroborating migration evidence from the Integrated Household Survey, the pre-famine period validation, and the spatial autocorrelation analysis collectively make this one of the more methodologically robust attempts to estimate the effect of a climate-related shock on HIV dynamics. However, the design cannot fully disentangle migration-driven compositional change in the antenatal population from true incidence-driven prevalence change. The observed decline in urban prevalence was attributed to dilution by low-risk rural migrants, even as those migrants' individual long-term infection risk nearly doubled likely due to social isolation and inequity. The inferential chain extending from drought-related increases in maternal HIV prevalence to vertical transmission risk introduces further uncertainty and depends on factors not quantified within the study, including maternal viral load, stage of infection, ART coverage, and breastfeeding practices. The biological dimension of this relationship merits separate consideration. Heat stress, nutritional deprivation, and physiological stress responses associated with drought and crop failure may impair cellular immune defences, increasing susceptibility to infection or accelerating disease progression in women living with HIV. Pregnant women, who are additionally immunomodulated, may be particularly vulnerable to these compounding effects. As climate variability intensifies across predominantly rain-fed agricultural regions of sub-Saharan Africa (47), the pathway linking food system disruption to HIV risk is likely to become increasingly consequential. Importantly, EWEs can interrupt supply chains for antiretroviral therapy and antenatal screening reagents, displace healthcare workers, and reduce women's capacity and willingness to attend antenatal care, PMTCT services, or intrapartum facilities (48). In settings where vertical transmission is prevented principally through clinical intervention, disruption to prevention services may represent the most direct route by which climate change translates into increased perinatal HIV transmission.

Ford et al. (40) documented a temporally coincident increase in bacterial meningitis incidence during a drought period in Eswatini. The authors suggested that this finding was due to drought-driven nutritional deterioration, overcrowding, and reduced immune competence. While this observation is contextually suggestive, the dominant identified organisms are not all vertically transmissible, and the causative organisms responsible for the drought-coincident spike remain unknown. Given this aetiological uncertainty and the absence of disaggregated age-specific data, this study contributes contextual plausibility to the broader hypothesis that EWE-related deprivation may increase susceptibility to serious neonatal and infant infection, but cannot be used to support conclusions specific to vertical transmission.

Taken together, the drought and famine evidence provides preliminary but methodologically limited support for a relationship between EWEs and infection outcomes in maternal and neonatal populations. These studies highlight important underlying drivers, converging on socioeconomic disruption, forced migration, and malnutrition, while also suggesting that EWE-driven resource scarcity can alter disease dynamics.

The observed directional pattern for VZV in Chan et al. (45) and Critselis et al. (41) is biologically plausible and consistent with broader literature. However, it is important to note that the climate–VZV associations identified in these studies operate through horizontal transmission rather than through direct modification of vertical transmission risk. The relevance to vertical transmission is therefore indirect as climate conditions that increase horizontal VZV transmission in the general population also increase the probability of primary VZV infection during pregnancy. VZV is an enveloped airborne pathogen whose stability in aerosols is sensitive to temperature and absolute humidity (49). Experimental evidence on enveloped airborne viruses demonstrates that viral survival time on surfaces and in droplet nuclei is substantially reduced at higher temperatures and humidity levels (50), this mechanism likely underlies the pronounced winter seasonality of VZV in temperate regions. Lower relative humidity reduces the density of air particles, allowing the virus to remain suspended longer and facilitating aerosolized transmission (51). Behavioural amplifiers reinforce this as more time spent indoors, altered hygiene practices, and poor ventilation increase contact rates and per-contact transmission probability (52, 53). A cross-national analysis has shown that VZV acquisition is markedly delayed in tropical and subtropical populations relative to temperate ones, with susceptibility persisting into adulthood in warmer and more humid regions (54). The seroprevalence gradient reported by Lolekha et al. (44) across Thai climate zones extends this evidence by highlighting the lower seroprevalence in warmer, more humid southern regions than in the cooler northern regions, a pattern that is consistent with the same underlying mechanism of temperature-and humidity-dependant inhibition of viral transmission operating across a climate gradient rather than a seasonal one. These findings may have important implications for the risk of VZV during pregnancy, since adult infection is associated with substantially higher rates of complications including varicella pneumonia (55). Additionally, in settings where cooler or drier conditions increase community VZV circulation, seronegative pregnant women face elevated risk of primary infection, with downstream risk of congenital varicella syndrome or neonatal varicella. The critical inferential gap is that no study has directly examined whether climate conditions modify the incidence of primary VZV infection in pregnant women or the subsequent risk of vertical transmission. The literature specifically addressing climate-related exposures, VZV in pregnancy, and neonatal outcomes is, on the basis of this review, essentially absent.

GBS is a Gram-positive commensal organism and a major cause of still birth, preterm birth and neonatal sepsis (25, 26). The vaginal and rectal carriage of GBS is influenced by local mucosal conditions. Experimental *in vitro* studies report significant increases in bacterial adherence at higher incubation temperatures following the same incubation period (56), suggesting a plausible cellular mechanism by which higher ambient temperatures, mediated through vaginal temperature, could increase colonisation density and prevalence. The findings of Dadvand et al. (43) are consistent with the broader literature on associations between warmer and more humid climates and streptococcal infections of the skin and soft tissues (57). Evidence shows that GBS colonisation rates vary substantially between high- and low-income settings (58), and across seasonal cycles within single populations (59, 60), though the climatic determinants of this variation have been poorly studied. While this study provides evidence to suggest that global warming could potentially contribute to the expansion of the maternal reservoir for neonatal sepsis, the colonisation-to-transmission gap is important. Maternal GBS colonisation is necessary but not sufficient for neonatal early-onset GBS disease. Transmission risk depends on availability of screening, intrapartum antibiotic prophylaxis (IAP) administration, and maternal colonisation density at delivery, among other factors (61). In settings with universal screening and high IAP coverage, a climate change driven increase in maternal GBS colonisation would only threaten to increase vertical transmission if the resulting downstream pressures on healthcare resources overwhelmed the capacity of screening and IAP programs absorb the rise in colonisation prevalence. Hence, the implications may be more relevant in high-burden settings without universal screening, however this remains unexplored.

The opposing directions of effect for VZV and GBS are themselves coherent, reflecting the fundamentally different transmission ecologies of an airborne pathogen whose aerosol stability is diminished by heat and humidity and a mucosal commensal bacterium whose carriage may be promoted by the same conditions. The evidence from this review suggests that the effects of climate change on infection dynamics may be pathogen-specific, rather than a single overarching direction of risk across all VTIs.

While no studies examined vertically transmissible vector-borne diseases, this represents an important gap rather than an absence of concern. Climate change alters vector ecology through rising temperatures, changing rainfall patterns, and shifting land use, driving the proliferation and geographic expansion of vector populations into previously unaffected territories (6). Zika virus can cause congenital Zika syndrome and its geographic range is projected to expand substantially under warming scenarios (62). Dengue virus has been associated with adverse pregnancy outcomes and potential vertical transmission, with vector range expansion already documented across Africa and Asia (63). Malaria remains one of the most significant causes of adverse maternal and neonatal outcomes globally, and climate-driven shifts in vector distribution threaten to extend transmission into higher-altitude and previously non-endemic regions of sub-Saharan Africa and South Asia (63). As the geographic footprint of these vectors expands, the pool of pregnant women at risk of acquiring and transmitting these infections to their fetuses or neonates will grow correspondingly, making this an urgent priority for future research and climate adaptation planning in maternal and neonatal health.

This review is, to our knowledge, the first systematic synthesis to examine a full spectrum of VTIs in relation to climate change in maternal and neonatal populations. This review's rationale is directly supported by an overview of systematic reviews of climate change and infectious diseases conducted by Conway et al. (2024), which identified the complete absence of existing syntheses addressing climate-sensitive infectious diseases, and maternal and newborn health (15).

The broader literature on climate change and infectious diseases has established associations between temperature, humidity, and EWEs, and a range of communicable diseases, including vector-borne infections, enteric diseases, and respiratory infections, with most evidence concentrated in non-pregnant general populations (5–8, 63, 64). This provides a comparative context for understanding the magnitude and consistency of climate change–infection associations, however findings may not be directly applicable to pregnant women or neonates given the immunological and physiological differences of these populations. Studies and reviews of vertically transmissible infections in maternal and neonatal populations that address HIV mother-to-child transmission (65), congenital syphilis (66), neonatal GBS sepsis (67, 68), and TORCH infections (68, 69), have characterised transmission rates, risk factors, and prevention strategies in varied settings, but do not address climate-related exposure variables. The present review bridges these two bodies of literature for the first time, identifying directionally coherent preliminary signals at the intersection of climate change and VTI risk, and characterising where the inferential gaps between existing evidence and clinical or policy relevance lie.

The limitations of this review must be considered when interpreting its findings. Searches were restricted to three databases and English-language publications, which may have missed relevant studies indexed in regional databases or published in other languages, particularly from high-burden regions in sub-Saharan Africa, South Asia, and Latin America. The heterogeneity of included study designs, exposure metrics, and outcome operationalisations precluded quantitative synthesis and limited direct cross-study comparison. Climate exposures were measured at the ecological level introducing exposure misclassification biases that may under- or over-estimate true associations. Critically, no included study was designed as a climate change attribution study, and none measured vertical transmission as a direct outcome; findings therefore reflect climate sensitivity evidence and infection outcomes used as proxies for vertical transmission risk, which introduces indirectness that is reflected in the very low certainty ratings across all outcome groupings. All included manuscripts were published prior to 2015, predating the emergence of formal climate attribution methods, limiting the ability to establish whether the associations identified in this review are attributable to anthropogenic climate change specifically, and likely reflects the genuinely nascent state of this research intersection rather than a search limitation. The absence of post-2015 studies represents an important evidence gap and a priority area for future research.

The findings of this review highlight specific research opportunities (Table 3). The GBS–temperature/humidity association warrants replication in diverse geographic and income settings, with follow-through to neonatal outcomes. Prospective cohort studies in pregnant women represent a particularly tractable opportunity for this kind of evidence generation, offering individual-level gestational climate exposure measurement alongside longitudinal GBS colonisation monitoring and neonatal infection outcomes. A high-burden sub-Saharan African or South Asian setting, where universal GBS screening is less common and prophylaxis coverage is lower, would directly test whether the climate–colonisation association translates into modified transmission risk in a clinically meaningful context. The HIV–drought association warrants prospective investigation at ANC surveillance sites in drought-vulnerable regions, incorporating PMTCT service coverage and viral load data to study pathways from EWEs to transmission risk rather than prevalence alone. For VZV, a prospective cohort with serological monitoring across seasons and climate zones would address the current absence of any maternal-population data on acquisition risk.

**Table 3:** Summary of recommendations for future research and clinical practice.

Domains		Recommendation
Research priorities	GBS	Replicate the GBS–temperature/humidity association in diverse geographic and income settings, with longitudinal follow-up to neonatal infection outcomes.
	HIV	Conduct prospective studies at ANC surveillance sites in drought-vulnerable regions, incorporating PMTCT coverage and viral load monitoring.
	VZV	Establish prospective cohorts with serological monitoring across seasons and climate zones in pregnant women
	Vector-borne VTIs	Investigate climate-driven shifts in vector-borne VTI risk (Zika, dengue, malaria) in pregnant populations in expanding transmission zones.
Methods	Exposure measurement	Prioritise individual-level gestational climate exposure measurement over ecological proxies.
	Outcome measurement	Use confirmed neonatal infection or vertical transmission as primary outcomes rather than maternal proxies.
	Study populations	Ensure representation of low- and middle-income settings in future studies.
	Modelling	Develop climate scenario models projecting future VTI burden and identifying high-risk geographies.
Practice	Health systems	Ensure supply chains, service delivery platforms, and healthcare worker capacity underpinning PMTCT, GBS screening, and STI treatment programmes are resilient to EWE-related disruption.

Future studies should give attention to a wider range of EWEs and must prioritise individual-level exposure measurement, longitudinal follow-up to neonatal infection outcomes, and representation of low- and middle-income settings where climate vulnerability, fragile healthcare systems, and infection burden converge. Complementary modelling studies could make an important contribution to this evidence base by projecting future VTI burden under different climate scenarios and identifying high-risk geographies where empirical research and targeted prevention efforts should be prioritised.

The very low certainty of the available evidence means that this review cannot support specific clinical practice changes or targeted public health interventions directed at climate change related VTI risk. However, the findings provide a scientifically coherent rationale for integrating climate vulnerability into existing frameworks for maternal and neonatal infectious disease prevention. Health systems should ensure that supply chains, service delivery platforms, and healthcare worker capacity underpinning PMTCT, GBS screening, and STI treatment programmes are resilient to disruption from EWEs. Until the evidence base matures, the most actionable policy implication of this review is that climate change should be recognised as a potential structural determinant of VTI risk.

## 5 Conclusion

In conclusion, this review found limited but suggestive evidence that climate-related exposures may be associated with infection outcomes relevant to vertical transmission through pathogen-specific biological pathways and broader social disruption associated with EWEs. However, the current evidence base remains too sparse, indirect, and methodologically weak to determine whether climate change is already altering vertical transmission and related neonatal outcomes in a measurable way. Closing this gap should be a priority for both research and climate adaptation in MNH.

## Data Availability

The original contributions presented in the study are included in the article/Supplementary Material, further inquiries can be directed to the corresponding author.

## References

[1] Patz JA Hatch MJ. Public health and global climate disruption. Public Health Rev. (2013) 35(1). 10.1007/BF0339169732226195

[2] World Meteorological Organization (WMO). State of the Global Climate 2025 (2026). Available online at: 10.59327/WMO/S/CRI/SOC1

[3] United Nations Environment Programme Emissions gap report 2025: Off target – Continued collective inaction puts global temperature goal at risk (2025). 10.59117/20.500.11822/48854

[4] IPCC. Summary for Policymakers. In: (2023) Climate Change 2023: Synthesis Report. Contribution of Working Groups I, II and III to the Sixth Assessment Report of the Intergovernmental Panel on Climate Change [Core Writing Team, H. Lee and J. Romero (eds.)] (2023). 10.59327/IPCC/AR6-9789291691647.001

[5] Moir M Baxter C Tegally H De Oliveira T. Infectious diseases in a warming world: a call for action. S Afr J Sci. (2025) 121(3/4). 10.17159/sajs.2025/20722

[6] Pavia G Branda F Ciccozzi A Romano C Locci C Azzena I. The issue of climate change and the spread of tropical diseases in Europe and Italy: vector biology, disease transmission, genome-based monitoring and public health implications. Infect Dis. (2025) 57:121–36. 10.1080/23744235.2024.2437027 39663537

[7] Bastug KA Schwartz EL Sam-Agudu NA. The impact of environmental changes on infectious diseases among children in North America. Curr Opin Pediatr. (2025) 37:153–64. 10.1097/MOP.0000000000001436 39916586

[8] Naga NG Taha RM Hamed EA Nawar EA Jaheen HO Mobarak AA. The silent microbial shift: climate change amplifies pathogen evolution, microbiome dysbiosis, and antimicrobial resistance. Trop Dis Travel Med Vaccines. (2025) 11:43. 10.1186/s40794-025-00275-y 41233921 PMC12616928

[9] Watts N Amann M Arnell N Ayeb-Karlsson S Beagley J Belesova K. The 2020 report of the lancet countdown on health and climate change: responding to converging crises. Lancet. (2021) 397:129–70. 10.1016/S0140-6736(20)32290-X 33278353 PMC7616803

[10] Stimpson JP Rashed AL Pandya J Baudot EC Whitfill J Ortega AN. Health equity in the wake of disasters and extreme weather: evidence from an umbrella review. Health Aff Scholar. (2025) 3:qxaf207. 10.1093/haschl/qxaf207 PMC12603904 41229451

[11] Ngcamu BS. Climate change effects on vulnerable populations in the Global South: a systematic review. Natural Hazards. (2023) 118:977–91. 10.1007/s11069-023-06070-2

[12] Roos N Kovats S Hajat S Filippi V Chersich M Luchters S. Maternal and newborn health risks of climate change: a call for awareness and global action. Acta Obstet Gynecol Scand. (2021) 100:566–70. 10.1111/aogs.14124 33570773

[13] Intergovernmental Panel on Climate Change (IPCC). Climate Change 2022 – Impacts, Adaptation and Vulnerability Cambridge: Cambridge University Press (2023). 10.1017/9781009325844

[14] Perera F Nadeau K. Climate change, fossil-fuel pollution, and children's health. N Engl J Med. (2022) 386:2303–14. 10.1056/NEJMra2117706 35704482

[15] Conway F Portela A Filippi V Chou D Kovats S. Climate change, air pollution and maternal and newborn health: an overview of reviews of health outcomes. J Glob Health. (2024) 14:04128. 10.7189/jogh.14.04128 38785109 PMC11117177

[16] Yüzen D Graf I Diemert A Arck PC. Climate change and pregnancy complications: from hormones to the immune response. Front Endocrinol (Lausanne). (2023) 14:1149284. 10.3389/fendo.2023.1149284 PMC10113645 37091849

[17] Cowell W Ard N Herrera T Medley EA Trasande L. Ambient temperature, heat stress and fetal growth: a review of placenta-mediated mechanisms. Mol Cell Endocrinol. (2023) 576:112000. 10.1016/j.mce.2023.112000 37460007

[18] Parnes MF Mosley L Burris HH Weiss EM. Climate change and environmental degradation: bioethical considerations and impact for neonatal care. Semin Perinatol. (2025) 49:152099 10.1016/j.semperi.2025.152099 40514319

[19] Basha S Surendran N Pichichero M. Immune responses in neonates. Expert Rev Clin Immunol. (2014) 10:1171–84. 10.1586/1744666X.2014.942288 25088080 PMC4407563

[20] National Library of Medicine. Infectious Disease Transmission, Vertical (MeSH scope note) (2009). Available online at: https://www.ncbi.nlm.nih.gov/mesh/?term=Infectious+Disease+Transmission%2C+Vertical (Accessed June 12 2026) 10.1080/15360280801989377 28792816

[21] Kumar M Saadaoui M A Khodor S. Infections and pregnancy: effects on maternal and child health. Front Cell Infect Microbiol. (2022) 12:873253. 10.3389/fcimb.2022.873253 35755838 PMC9217740

[22] Lian J Ke B Zhang X Li J Li Y Wang K. Global burden of HIV, syphilis, and HBV infection among women of childbearing age and children under five: based on the global burden of diseases study 2021. Front Public Health. (2025) 13:1666408. 10.3389/fpubh.2025.1666408 41179781 PMC12571732

[23] UNAIDS. Fact sheet - Latest global and regional statistics on the status of the AIDS epidemic (2025)

[24] World Health Organization. Syphilis [Factsheet] (2025)

[25] Mei JY Silverman NS. Group B streptococcus in pregnancy. Obstet Gynecol Clin North Am. (2023) 50:375–87. 10.1016/j.ogc.2023.02.009 37149317

[26] Seale AC Bianchi-Jassir F Russell NJ Kohli-Lynch M Tann CJ Hall J. Estimates of the burden of group B streptococcal disease worldwide for pregnant women, stillbirths, and children. Clin Infect Dis. (2017) 65:S200–19. 10.1093/cid/cix664 29117332 PMC5849940

[27] Madrid L Varo R Sitoe A Bassat Q. Congenital and perinatally-acquired infections in resource-constrained settings. Expert Rev Anti Infect Ther. (2016) 14:845–61. 10.1080/14787210.2016.1215913 27442227

[28] Helldén D Andersson C Nilsson M Ebi KL Friberg P Alfvén T. Climate change and child health: a scoping review and an expanded conceptual framework. Lancet Planet Health. (2021) 5:e164–75. 10.1016/S2542-5196(20)30274-6 33713617

[29] Orievulu KS Ayeb-Karlsson S Ngema S Baisley K Tanser F Ngwenya N. Exploring linkages between drought and HIV treatment adherence in Africa: a systematic review. Lancet Planet Health. (2022) 6:e359–70. 10.1016/S2542-5196(22)00016-X 35397224 PMC7612934

[30] Iwuji CC McMichael C Sibanda E Orievulu KS Austin K Ebi KL. Extreme weather events and disruptions to HIV services: a systematic review. Lancet HIV. (2024) 11:e843–60. 10.1016/S2352-3018(24)00186-3 39393367

[31] Lieber M Chin-Hong P Whittle HJ Hogg R Weiser SD. The synergistic relationship between climate change and the HIV/AIDS epidemic: a conceptual framework. AIDS Behav. (2021) 25:2266–77. 10.1007/s10461-020-03155-y 33452659 PMC7810285

[32] Page MJ McKenzie JE Bossuyt PM Boutron I Hoffmann TC Mulrow CD. The PRISMA 2020 statement: an updated guideline for reporting systematic reviews. Br Med J. (2021) 372:n71. 10.1136/bmj.n71 PMC8005924 33782057

[33] Campbell M McKenzie JE Sowden A Katikireddi SV Brennan SE Ellis S. Synthesis without meta-analysis (SWiM) in systematic reviews: reporting guideline. Br Med J. (2020) 368:l6890. 10.1136/bmj.l6890 PMC7190266 31948937

[34] Moola S Munn Z Tufanaru C Aromataris E Sears K Sfetcu R. Chapter 7: Systematic reviews of etiology and risk. In: Aromataris E Lockwood C Porritt K Pilla B Jordan Z, editors. JBI Manual for Evidence Synthesis, North Adelaide, SA: JBI, School of Public Health, Adelaide University, (2020), 296–310.

[35] Barker TH Hasanoff S Aromataris E Stone JC Leonardi-Bee J Sears K. The revised JBI critical appraisal tool for the assessment of risk of bias for analytical cross-sectional studies. JBI Evid Synth. (2026) 24:401–8. 10.11124/JBIES-24-00523 40521701

[36] Munn Z Moola S Lisy K Riitano D Tufanaru C. Chapter 5: systematic reviews of prevalence and incidence. JBI Manual for Evidence Synthesis. (2020). 10.46658/JBIRM-17-05

[37] Bhaskaran K Gasparrini A Hajat S Smeeth L Armstrong B. Time series regression studies in environmental epidemiology. Int J Epidemiol. (2013) 42:1187–95. 10.1093/ije/dyt092 23760528 PMC3780998

[38] McGuinness LA Higgins JPT. Risk-of-bias VISualization (robvis): an R package and shiny web app for visualizing risk-of-bias assessments. Res Synth Methods. (2021) 12:55–61. 10.1002/jrsm.1411 32336025

[39] Schünemann HJ Brennan S Akl EA Hultcrantz M Alonso-Coello P Xia J. The development methods of official GRADE articles and requirements for claiming the use of GRADE – A statement by the GRADE guidance group. J Clin Epidemiol. (2023) 159:79–84. 10.1016/j.jclinepi.2023.05.010 37211327

[40] Ford H Wright J Shepherd G Africa Ford J Wright SH Yorkshire Health Authority N. Bacterial meningitis in Swaziland: an 18 month prospective study of its impact. J Epidemiol Community Health. (1994) 48:276–80. 10.1136/jech.48.3.276 8051527 PMC1059959

[41] Critselis E Nastos PT Theodoridou K Theodoridou M Tsolia MN Hadjichristodoulou C. Time trends in pediatric hospitalizations for Varicella infection are associated with climatic changes: a 22-year retrospective study in a tertiary Greek referral center. PLoS One. (2012) 7:e52016. 10.1371/journal.pone.0052016 23284855 PMC3532345

[42] Loevinsohn M. The 2001−03 famine and the dynamics of HIV in Malawi: a natural experiment. PLoS One. (2015) 10:e0135108. 10.1371/journal.pone.0135108 26332405 PMC4558031

[43] Dadvand P Basagana X Figueras F Sunyer J Nieuwenhuijsen MJ. Climate and group B streptococci colonisation during pregnancy: present implications and future concerns. BJOG. (2011) 118:1396–400. 10.1111/j.1471-0528.2011.03044.x 21749630

[44] Lolekha S Tanthiphabha W Sornchai P Kosuwan P Sutra S Warachit B. Effect of climatic factors and population density on varicella zoster virus epidemiology within a tropical country. Am J Trop Med Hyg. (2001) 64:131–6. 10.4269/ajtmh.2001.64.131 11442207

[45] Chan JYC Tian L Kwan YW Chan WM Leung CW. Hospitalizations for varicella in children and adolescents in a referral hospital in Hong Kong, 2004 to 2008: a time series study. BMC Public Health. (2011) 11:366. 10.1186/1471-2458-11-366 21605371 PMC3119164

[46] De Vocht F Katikireddi SV McQuire C Tilling K Hickman M Craig P. Conceptualising natural and quasi experiments in public health. BMC Med Res Methodol. (2021) 21:32. 10.1186/s12874-021-01224-x 33573595 PMC7879679

[47] Omokpariola DO Agbanu-Kumordzi C Samuel T Kiswii L Moses GS Adelegan AM. Climate change, crop yield, and food security in sub-saharan Africa. Discov Sustain. (2025) 6:1–21. 10.1007/s43621-025-01580-4

[48] Haq SMA Mawa J Tasneem N Saha B Ahmed MNQ Ahmed KJ. Nexus between climate change and maternal mortality: a review of literature. SN Social Sciences. (2025) 5:175. 10.1007/s43545-025-01212-8

[49] Dempsey SJ Andersen LK Coates SJ Davis MDP. Impact of climatic factors on varicella-zoster viral infection. Int J Dermatol Venereol. (2025) 8:61–2. 10.1097/JD9.0000000000000347

[50] Biryukov J Boydston JA Dunning RA Yeager JJ Wood S Reese AL. Increasing temperature and relative humidity accelerates inactivation of SARS-CoV-2 on surfaces. mSphere. (2020) 5:e00441–20 10.1128/msphere.00441-20 32611701 PMC7333574

[51] Skanata A Spagnolo F Metz M Smyth DS Dennehy JJ. Humidity reduces rapid and distant airborne dispersal of viable viral particles in classroom settings. Environ Sci Technol Lett. (2022) 9:632–7. 10.1021/acs.estlett.2c00243 35937034 PMC9344459

[52] Banholzer N Munday JD Jent P Bittel P Dall'Amico L Furrer L. The relative contribution of close-proximity contacts, shared classroom exposure and indoor air quality to respiratory virus transmission in schools. Nat Commun. (2025) 16:11678. 10.1038/s41467-025-66719-3 41309642 PMC12749238

[53] Moriyama M Hugentobler WJ Iwasaki A. Seasonality of respiratory viral infections. Annu Rev Virol. (2020) 7:83–101. 10.1146/annurev-virology-012420-022445 32196426

[54] Lee. Review of varicella zoster seroepidemiology in India and south-east Asia. Trop Med Int Health. (1998) 3:886–90. 10.1046/j.1365-3156.1998.00316.x 9855401

[55] Alanezi M. Varicella pneumonia in adults: 13 years' Experience with review of literature. Ann Thorac Med. (2007) 2:163–5. 10.4103/1817-1737.36551 19727368 PMC2732098

[56] Zawaneh SM Ayoub EM Baer H Cruz AC Spellacy WN. Factors influencing adherence of group B streptococci to human vaginal epithelial cells. Infect. Immun. (1979) 26(2). 10.1128/iai.26.2.441-447.1979 44701 PMC414634

[57] Bisno AL Stevens DL. Streptococcal infections of skin and soft tissues. N Engl J Med. (1996) 334:240–6. 10.1056/NEJM199601253340407 8532002

[58] Gonçalves BP Procter SR Paul P Chandna J Lewin A Seedat F. Group B streptococcus infection during pregnancy and infancy: estimates of regional and global burden. Lancet Glob Health. (2022) 10:e807–19. 10.1016/S2214-109X(22)00093-6 PMC9090904 35490693

[59] L Doare K Jarju S Darboe S Warburton F Gorringe A Heath PT. Risk factors for group B Streptococcus colonisation and disease in gambian women and their infants. J Infect. (2016) 72:283–94. 10.1016/j.jinf.2015.12.014 26763186 PMC4769314

[60] Paveenkittiporn W Ungcharoen R Kerdsin A. Streptococcus agalactiae infections and clinical relevance in adults, Thailand. Diagn Microbiol Infect Dis. (2020) 97:115005. 10.1016/j.diagmicrobio.2020.115005 32081521

[61] ACOG Committee. Prevention of group B streptococcal early-onset disease in newborns. Obstet Gynecol. (2020) 135:e51–72. 10.1097/AOG.0000000000003668 31977795

[62] Van Wyk H Eisenberg JNS Brouwer AF. Long-term projections of the impacts of warming temperatures on Zika and dengue risk in four Brazilian cities using a temperature-dependent basic reproduction number. PLoS Negl Trop Dis. (2023) 17:e0010839. 10.1371/journal.pntd.0010839 37104296 PMC10138270

[63] Kulkarni MA Duguay C Ost K. Charting the evidence for climate change impacts on the global spread of malaria and dengue and adaptive responses: a scoping review of reviews. Global Health. (2022) 18:1. 10.1186/s12992-021-00793-2 34980187 PMC8725488

[64] Nzekwe JC. Shifting vector ecologies in a warming world: implications of climate change for infectious disease and healthcare systems. Int J Biol Sci. (2025) 7:135–46. 10.33545/26649926.2025.v7.i10b.508

[65] Thorne C Newell M-L. Prevention of mother-to-child transmission of HIV infection. Curr Opin Infect Dis. (2004) 17:247–52. 10.1097/00001432-200406000-00013 15166829

[66] Zhang X Yu Y Yang H Xu H Vermund SH Liu K. Surveillance of maternal syphilis in China: pregnancy outcomes and determinants of congenital syphilis. Med Sci Monit. (2018) 24:7727–35. 10.12659/MSM.910216 30370903 PMC6216479

[67] Coggins SA Puopolo KM. Neonatal group B Streptococcus disease. Pediatr Rev. (2024) 45:63–73. 10.1542/pir.2023-006154 38296778 PMC10919294

[68] Puopolo KM. Epidemiology of neonatal early-onset sepsis. Neoreviews. (2008) 9:e571–9. 10.1542/neo.9-12-e571 36316253

[69] Pavia G Licata F Marascio N Giancotti A Tassone MT Costa C. Seroprevalence and age-related susceptibility of TORCH infections in childbearing age women: a 5-year cross-sectional retrospective study and a literature review. J Infect Public Health. (2024) 17:102537. 10.1016/j.jiph.2024.102537 39255545

