# Supplementary material for: Climate change and vertically transmissible infections in maternal and neonatal populations: a systematic review
Source: Front Glob Womens Health. 2026 Sep 23;7:1931882. doi: 10.3389/fgwh.2026.1931882 (PMC13642628; doi:10.3389/fgwh.2026.1931882)
Supplement: Supplementary file 1 [file Table1.docx]

**Climate change and vertically transmissible infections in maternal and neonatal populations: a systematic review**

**Supplementary material**

[Supplementary Table 1. Characteristics and reasons for exclusion of full-text studies assessed for eligibility (n=36)	1](#_Toc235624578)

[Supplementary Table 2. Summary of findings with certainty of evidence (GRADE)	3](#_Toc235624579)

[Database Search Strategies	4](#_Toc235624580)

[1. PubMed	4](#_Toc235624581)

[2. Web of Science	6](#_Toc235624582)

[3. Scopus	7](#_Toc235624583)

# **Supplementary Table 1.** Characteristics and reasons for exclusion of full-text studies assessed for eligibility (n=36)

| First Author (Year) | Title | Pathogen/Topic | Reason for Exclusion |
| --- | --- | --- | --- |
| Asad (2018) | Effects of climate change on the spread of Zika virus: a public health threat | Zika virus | Incorrect study design |
| Atai (2025) | Zika virus in malaria-endemic populations: a climate change-driven syndemic in the Sudan Savannah, Nigeria | Zika virus | Incorrect/missing exposure |
| Bizimana (2015) | Assessing the social vulnerability to malaria in Rwanda | Malaria | Incorrect study design |
| Bojar (2010) | Environmental exposure of pregnant women to infection with Toxoplasma gondii | Toxoplasmosis | Incorrect study design |
| Buekens (2024) | Preparing for the next emerging perinatal infectious disease | Perinatal infections | Incorrect study design |
| Chan (2016) | Zika fever and congenital Zika syndrome: an unexpected emerging arboviral disease | Zika virus | Incorrect study design |
| de Lima (2021) | Intelligent systems for dengue, chikungunya, and Zika temporal and spatio-temporal forecasting | Dengue/chikungunya/Zika | Incorrect study design |
| Douglas (2024) | The silent invaders: Oropouche and Melao viruses, causes of increased public health risks for the Americas | Oropouche/Melao virus | Incorrect study design |
| Doyle (2023) | Seasonal patterns in newborns' health: quantifying the roles of climate, communicable disease, economic and social factors | Newborn health/infections | Incorrect/missing exposure |
| Gardini Sanches Palasio (2023) | Zika, chikungunya and co-occurrence in Brazil: space-time clusters and associated environmental-socioeconomic factors | Zika/chikungunya | Incorrect patient population |
| Githinji (2014) | Compound vulnerabilities: the intersection of climate variability and HIV/AIDS in northwestern Tanzania | HIV | Incorrect patient population |
| Hopkins (2022) | Chikungunya encephalitis: an inconsistently reported headache and cause of death in patients with pre-existing conditions | Chikungunya | Incorrect study design |
| Jones (2025) | Ecologic study learning module: Gordon et al. (2023), disparities in preterm birth following the July 1995 Chicago heat wave | Preterm birth | Incorrect study design |
| Lindtjorn (1992) | Child health and nutrition: a study from drought prone areas in southern Ethiopia | Child nutrition/health | Incorrect outcomes |
| Logie (2024) | HIV vulnerabilities associated with water insecurity, food insecurity, and other COVID-19 impacts among urban refugee youth in Kampala, Uganda | HIV | Incorrect patient population |
| Lorea (2025) | Infections during pregnancy: an ongoing threat | Pregnancy infections | Incorrect study design |
| Mancini (2021) | High temperature cycles result in maternal transmission and dengue infection differences between Wolbachia strains in Aedes aegypti | Dengue | Incorrect patient population |
| Mason (2005) | AIDS, drought, and child malnutrition in southern Africa | HIV/malnutrition | Incorrect outcomes |
| Mason (2010) | Impact of drought and HIV on child nutrition in eastern and southern Africa | HIV/nutrition | Incorrect outcomes |
| Oberlin (2023) | Vector-borne disease, climate change and perinatal health | Vector-borne infections | Incorrect study design |
| Onozuka (2011) | Effect of weather variability on the incidence of mumps in children: a time-series analysis | Mumps | Incorrect outcomes |
| Oscar Lipp (2018) | Climate change, air pollution, and infectious diseases: a new epidemiological scenario in Argentina | Infectious diseases | Incorrect study design |
| Palmer (2024) | Food and water insecurity in households of children and adolescents living with HIV and receiving care in a rural Zambian hospital | HIV | Incorrect patient population |
| Procianoy (2025) | Impact of the May 2024 floods on childhood immunization coverage in Rio Grande do Sul, Brazil | Immunisation coverage | Incorrect outcomes |
| Rasmussen (2022) | Protecting pregnant people and babies from the health effects of climate change | Maternal/neonatal health | Incorrect study design |
| Renzaho (2007) | Mortality rates, prevalence of malnutrition, and prevalence of lost pregnancies among the drought-ravaged population of Tete Province, Mozambique | Pregnancy outcomes | Incorrect patient population |
| Runkle (2023) | Climate change impacts on maternal health in a southern birth cohort: a causal analysis | Maternal health | Incorrect outcomes |
| Sarker (2025) | Childhood diarrheal infections among the multi-hazard disaster risk zones in Bangladesh | Diarrhoeal infections | Incorrect outcomes |
| Siddiqi (2025) | Assessing the impact of heat waves on childhood immunization coverage in Sindh, Pakistan | Immunisation coverage | Incorrect outcomes |
| Tlou (2018) | Underlying determinants of maternal mortality in a rural South African population with high HIV prevalence (2000–2014) | HIV/maternal mortality | Incorrect/missing exposure |
| Treibich (2022) | From a drought to HIV: an analysis of the effect of droughts on transactional sex and sexually transmitted infections in Malawi | HIV/STIs | Incorrect patient population |
| Tseng (2005) | A seroepidemiology study of varicella among children aged 0–12 years in Taiwan | VZV | Incorrect/missing exposure |
| Waseem (2025) | Reproductive health crisis amidst a natural disaster in Pakistan: a call to action | Reproductive health | Incorrect study design |
| Wiemers (2025) | Mothers and mosquitoes: climate change contributes to the spread of vector-borne pathogens posing a substantial threat to pregnant women | Vector-borne infections | Incorrect study design |
| Williams (2017) | Doctoral dissertation research: the role of thermoregulation in the obstetrical dilemma | Thermoregulation | Incorrect outcomes |
| Xu (2022) | Assessing the risk of spread of Zika virus under current and future climate scenarios | Zika virus | Incorrect patient population |

# **Supplementary Table 2.** Summary of findings with certainty of evidence (GRADE)

| **Outcome (Exposure → Infection → Population)** | **No. of studies (Design)** | **No. of participants** | **Relative effect (95% CI)** | **Anticipated absolute effects without exposure (comparator)** | **Anticipated absolute effects with exposure** | **Certainty of evidence (GRADE)** | **Importance** | **Basis for downgrading and key comments** |
| --- | --- | --- | --- | --- | --- | --- | --- | --- |
| **OUTCOME GROUP 1: Drought & famine → HIV prevalence in pregnant women** | | | | | | | | |
| HIV prevalence  Exposure: District-level famine severity (proportion of rural households requiring food assistance, Dec 2002–Mar 2003)  Population: Pregnant women aged 15–24 years attending ANC, Malawi Comparison: Pre-famine (1999/2000) vs famine/post-famine (2003) | 1 study (Natural experiment) | ~6,437 women (1999) ~7,705 women (2003) across multiple ANC sentinel sites | % change in HIV prevalence at rural sites: +20.2% (p=0.016) Non-linear relationship: no increase below ~25% district hunger threshold; steep increase above threshold.  Relative risk at sites with highest hunger: approximately 2.39. Adjusted for: age, occupation, education, site type, baseline HIV prevalence; random intercepts at site level. | Pre-famine rural HIV prevalence at ANC sites: data available at sentinel sites (1999/2000); absolute prevalence varies by site.  Non-rural sites showed decline (−21.7% semi-urban, −14.8% urban, p=0.006) consistent with migration of lower-prevalence rural women to cities. | 20.2% relative increase in HIV prevalence at rural ANC sites over the famine period, non-linearly associated with district hunger severity.  Effect concentrated among rural farming women attending ANC. Corroborated by independent migration data from IHS 2004–05. Spatial autocorrelation of residuals not significant at any distance. | ⊕◯◯◯ VERY LOW  Downgraded: −1 Indirectness (serious)  Not downgraded for RoB (moderate), inconsistency (N/A), or imprecision (no serious concern) | Critical | RoB: moderate — natural experiment with strong methodological safeguards. Multilevel modelling, adjustment for baseline prevalence, pre-famine period validation, spatial autocorrelation testing, sensitivity analyses. Limitations: ecological district-level exposure assignment; outcome is prevalence not incidence (reflects new infections + mortality + migration); only 8 rural sentinel sites; handling of site attrition by exclusion rather than sensitivity analysis.  Inconsistency: N/A — single study.  Indirectness (serious): Outcome is HIV prevalence in pregnant women, not VTR or neonatal infection. The gap between maternal HIV prevalence & VT depends on ART access, viral load, PMTCT coverage, & breastfeeding practices — none of which are reported. Drought operates through multiple mediating pathways, not direct biological effects on HIV virulence or maternal immune function. Long inferential chain to VTR.  Imprecision: No serious concern — large dataset, significant association, corroborated by four independent lines of evidence.   Publication bias: Some concern — single study in unique natural experiment context; direct replication structurally impossible.  Dose–response gradient: Non-linear threshold relationship present. Noted but cannot offset serious indirectness.  Starting certainty: Low.  Final: Very low. |
| **OUTCOME GROUP 2: Ambient temperature & humidity → Maternal GBS colonisation during pregnancy** | | | | | | | | |
| Maternal GBS colonisation (prevalence)  Exposure: Mean daily temperature, gestational weeks 32–36  Population: Full-term pregnant women, Barcelona, Spain (n=7,976) Study period: January 2001–June 2005 | 1 study (Retrospective cohort) | 7,976 women  1,359 GBS positive; prevalence 17.0% | Continuous:  aOR 1.02 per 1°C (95% CI 1.01–1.03; p=0.012)  Quartile comparison (vs Q1 <12.0°C):   - Q2 (12.0–15.4°C): aOR 1.01 (0.85–1.21) - Q3 (15.4–21.8°C): aOR 1.18 (0.99–1.40) - Q4 (>21.8°C): aOR 1.21 (1.02–1.45)   Adjusted for: age, ethnicity, education, employment, smoking, diabetes, parity | Reference: lowest temperature quartile (<12.0°C). GBS prevalence not reported by quartile in absolute terms; overall prevalence 17.0%.  August (peak summer) vs January (coldest): aOR 1.38 (95% CI 1.04–1.82). | 21% higher odds of GBS colonisation comparing hottest vs coolest month. Dose–response relationship suggestive for temperature (ORs 1.01, 1.18, 1.21 across quartiles) but only Q4 statistically significant. Exposure window selected empirically (lowest AIC) rather than a priori. | ⊕◯◯◯ VERY LOW  Downgraded: −1 Indirectness (serious) −1  Publication bias (serious)  Not downgraded for RoB (low), inconsistency (N/A), or imprecision (no serious concern) | Critical | RoB: low — strongest study in review. GIS-based individual exposure assignment from three meteorological stations. Validated microbiological outcome. Multivariable adjustment. Sensitivity analyses for preterm exclusion & seasonality (ANOVA p = 0.098). Main concern: data-driven exposure window selection (AIC-based). Antibiotic use during pregnancy not adjusted for.  Inconsistency: N/A (single study).  Indirectness (serious): Outcome is maternal colonisation, not neonatal GBS infection or VTR. Colonisation is necessary but not sufficient for neonatal disease; intrapartum antibiotic prophylaxis may mediate the gap. Study restricted to Barcelona— limited generalisability to high-burden LMIC settings. PTBs excluded.  Imprecision: No serious concern. CIs fully reported, quartile comparisons, effect sizes moderate & consistent  Publication bias (serious): Single study from one centre. No replication in other settings identified and authors note no prior study on this link. Risk of non-publication of null findings cannot be assessed.  Starting certainty: Low.  Final: Very low. |
| Maternal GBS colonisation (prevalence)  Exposure: Mean daily relative humidity (%), gestational weeks 32–36  Population: Full-term pregnant women, Barcelona, Spain (n=7,976) Study period: January 2001–June 2005 | 1 study (Retrospective cohort) | 7,976 women (1,359 GBS positive; prevalence 17.0%) | Continuous:  aOR 1.03 per 1% RH (95% CI 1.01–1.05; p=0.001)  Quartile comparison (vs Q1 <72.7%):   - Q2 (72.7–75.1%): aOR 1.30 (95% CI 1.09–1.56; p=0.004) - Q3 (75.1–77.3%): aOR 1.30 (95% CI 1.09–1.56; p=0.003) - Q4 (>77.3%): aOR 1.34 (95% CI 1.12–1.61; p=0.002)   Adjusted for: age, ethnicity, education, employment, smoking, diabetes, parity | Reference: lowest humidity quartile (<72.7%). Overall GBS prevalence 17.0%. Absolute prevalence by humidity quartile not separately reported. | 34% higher odds of GBS colonisation in highest vs lowest humidity quartile. More consistent dose–response across quartiles than for temperature (ORs 1.30, 1.30, 1.34 — all significant). Humidity effect robust across continuous & categorical analyses. | ⊕◯◯◯ VERY LOW  Downgraded: −1 Indirectness (serious) −1  Publication bias (serious) | Critical | RoB: low — same study as Outcome 2a.   Inconsistency: N/A — single study.  Indirectness (serious): Same as Outcome 2a   Imprecision: same study as Outcome 2a.  Publication bias: Same as Outcome 2a.  Dose–response gradient: Present & more consistent than for temperature (monotonic from Q1 reference through Q2 1.30, Q3 1.30, Q4 1.34, all p<0.005). Biologically plausible — warm, moist conditions may promote GBS mucosal carriage. Gradient noted but cannot offset serious downgrading for indirectness & publication bias.  Starting certainty: Low.  Final: Very low. |
| **OUTCOME GROUP 3: Ambient temperature & humidity → VZV infection outcomes in paediatric populations including infants** | | | | | | | | |
| VZV hospitalisation incidence  Exposure: Lower monthly relative humidity (lag 0)  Population: Children <18 yrs (including infants), Hong Kong Study period: 2004–2008 | 1 study (Retrospective time-series) | 598 hospitalisations over 60 months | β=−2.67 (95% CI −4.34 to −1.01) Poisson GEE regression Unit: log change in monthly count per unit increase in humidity (%) | Cool season reference: monthly VZV hospitalisations positively autocorrelated with prior month count (β lag₋₁=0.038, 95% CI 0.019–0.057) | Inverse association: lower relative humidity → higher monthly VZV hospitalisation count. Effect significant in cool season (β=−3.02, 95% CI −5.47 to −0.58, p=0.015); not significant in hot season. | ⊕◯◯◯ VERY LOW  Downgraded: −1 RoB (serious) −1 Indirectness (serious) −1 Imprecision (serious) | Critical | RoB(serious): Unaddressed time-varying confounders. No demographic adjustment. Autocorrelation modelled but seasonal confounding only partially controlled.  Indirectness (serious): Infants not disaggregated from broader paediatric cohort. Outcome is hospitalisation count, not VTR.   Imprecision (serious): CIs reported only for significant findings. Temperature & rainfall Spearman correlations lack precision measures.  Starting certainty: Low . Final: Very low. |
| VZV hospitalisation incidence  Exposure: Monthly air temperature, relative humidity, wind speed  Population: Children ≤14 years (incl. neonates & infants), Athens, Greece Study period: 1982–2003 | 1 study (Retrospective time-series) | 2,366 hospitalisations over 264 months | Temperature:  β=−0.030 (SE 0.008, p<0.001)  Wind speed:  β=+0.181 (SE 0.070, p=0.009)  Humidity:  β=−0.001 (SE 0.005, p=0.916) | Summer proportion of cases: increasing trend +0.49% per year (p=0.025) over 22 years.  Autumn proportion: decreasing −0.56% per year (p=0.021).  Spring: highest absolute burden (>40% of cases). | Secular shift towards more summer-season cases, interpreted as consistent with regional warming. Temperature association lost significance after seasonal adjustment.  Wind speed independently associated after adjustment. Humidity not independently significant. | ⊕◯◯◯ VERY LOW  Downgraded: −1 RoB (serious) −1 Indirectness (serious) −1 Imprecision (serious) | Critical | RoB (serious): 22-year period without adjustment for vaccination introduction, secular changes in admission practice, or population immunity. No model diagnostic checks reported. ARIMA parameters not stated. Absence of sensitivity analyses for secular trend findings.  Indirectness (serious): No infant-specific or neonatal estimates. Outcome is hospitalisation, not VTR or congenital VZV. No pregnant women enrolled.  Imprecision (serious): CIs absent throughout; SE only reported. Seasonal trend findings have no precision estimates.  Starting certainty: Low. Final: Very low. |
| VZV seroprevalence  Exposure: Regional climate zone (temperature, humidity, rainfall)  Population: Children 9 months–4 years, four Thai regions Study period: July 1997–April 1998 | 1 study (Cross-sectional seroepidemiological) | 2,093 participants  412 aged 9 mo. –4 yr across four regions | Regional seroprevalence (9 mo.–4 yr):   - North (coolest): 33.3% (95% CI 24.3–43.3) - NE (seasonal): 12.0% (95% CI 6.4–20.0) - Central (warm): 11.3% (95% CI 5.8–19.4) - South (warmest): 6.2% (95% CI 2.5–18.5) | Coolest region (Northern, ~10°C minimum temperature): seroprevalence 33.3% (95% CI 24.3–43.3) in youngest age group. | Consistent monotonic gradient: seroprevalence lowest in warmest regions & highest in coolest region (6.2% → 33.3% with decreasing temperature), suggesting high temperature & humidity inhibit VZV transmission & delay natural infection.  Urban/ rural residence & prior VZV history also significant predictors. | ⊕◯◯◯ VERY LOW  Downgraded: −1 RoB (moderate–serious) −1 Indirectness (serious) −1 Imprecision (serious) | Critical | RoB (moderate to serious): Volunteer convenience sampling with differing recruitment across regions introduces potential selection bias. Climate exposure assigned at regional level (ecological misclassification). Population density addressed via urban/rural proxy in models.  Indirectness (serious): Seroprevalence is a proxy for cumulative infection exposure, not incident VZV or VT. No maternal or neonatal outcomes. Long inferential chain to VTR.  Imprecision (serious): No formal tests of between-region differences reported for youngest age group. Wide CIs in Southern region (2.5–18.5%).  Funding: SmithKline Beecham Biologicals — potential conflict of interest.  Dose–response gradient insufficient to offset serious downgrading on other domains.  Starting certainty: Low.  Final: Very low. |

Abbreviations: aOR = adjusted odds ratio; ANC = antenatal clinic; ART = antiretroviral therapy; CI = confidence interval; GBS = group B streptococcus; GEE = generalised estimating equations; GBD = Global Burden of Disease; IHS = Integrated Household Survey; LMIC = low- and middle-income country; PMTCT = prevention of mother-to-child transmission; RH = relative humidity; VTR = vertical transmission rate; VZV = varicella-zoster virus; YLD = years lived with disability.

# **Database Search Strategies**

## **1. PubMed**

( "climate change"[Mesh] OR "global warming"[Mesh] OR "greenhouse effect"[Mesh] OR

"climate change"[tiab] OR "global warming"[tiab] OR "greenhouse effect"[tiab] OR

heatwave*[tiab] OR "heat wave*"[tiab] OR heat*[tiab] OR "hot temperature"[tiab] OR

thermal[tiab] OR drought*[tiab] OR "Droughts"[Mesh] OR flood*[tiab] OR

"Floods"[Mesh] OR wildfire*[tiab] OR "Wildfires"[Mesh] OR "extreme weather"[tiab] OR

"extreme rainfall"[tiab] )

AND

( "Pregnancy"[Mesh] OR "Pregnant People"[Mesh] OR pregnancy[tiab] OR pregnant[tiab] OR

maternal[tiab] OR antenatal[tiab] OR prenatal[tiab] OR intrapartum[tiab] OR

peripartum[tiab] OR postpartum[tiab] OR obstetric*[tiab] OR mother*[tiab] OR

neonatal[tiab] OR neonate*[tiab] OR newborn*[tiab] OR perinatal[tiab] OR infant*[tiab] )

AND

( "HIV"[Mesh] OR "HIV Infections"[Mesh] OR HIV[tiab] OR "human immunodeficiency virus"[tiab] OR

AIDS[tiab] OR "acquired immunodeficiency syndrome"[tiab] OR PLHIV[tiab] OR

"mother-to-child transmission"[tiab] OR MTCT[tiab] OR "vertical transmission"[tiab] OR

"perinatal transmission"[tiab] OR "Sexually Transmitted Diseases"[Mesh] OR

"sexually transmitted infection"[tiab] OR STI[tiab] OR STIs[tiab] OR STD[tiab] OR

STDs[tiab] OR "venereal disease"[tiab] OR syphilis[tiab] OR "Syphilis"[Mesh] OR

gonorrh*ea[tiab] OR "Gonorrh*ea"[Mesh] OR chlamydia[tiab] OR "Chlamydia Infections"[Mesh] OR

trichomonas*[tiab] OR "Trichomonas Infections"[Mesh] OR "Trichomonas Vaginitis"[Mesh] OR

"herpes simplex"[tiab] OR HSV[tiab] OR "Herpes Genitalis"[Mesh] OR

"human papillomavirus"[tiab] OR HPV[tiab] OR "Papillomavirus Infections"[Mesh] OR

"Mycoplasma genitalium"[tiab] OR "Pelvic Inflammatory Disease"[tiab] OR

"Pelvic Inflammatory Disease"[Mesh] OR PID[tiab] OR "Toxoplasmosis, Congenital"[Mesh] OR

toxoplasmosis[tiab] OR "Toxoplasma gondii"[tiab] OR "Rubella"[Mesh] OR rubella[tiab] OR

"Rubella Syndrome, Congenital"[Mesh] OR "Cytomegalovirus Infections"[Mesh] OR

cytomegalovirus[tiab] OR CMV[tiab] OR "Varicella Zoster Virus Infection"[Mesh] OR

"Herpesvirus 3, Human"[Mesh] OR "Chickenpox"[Mesh] OR varicella[tiab] OR

chickenpox[tiab] OR "Varicella-Zoster Virus"[tiab] OR "Parvovirus B19, Human"[Mesh] OR

parvovirus[tiab] OR "Parvovirus B19"[tiab] OR "Zika Virus Infection"[Mesh] OR

zika[tiab] OR "Streptococcus agalactiae"[Mesh] OR "group B streptococcus"[tiab] OR

GBS[tiab] OR "Hepatitis B"[Mesh] OR "Hepatitis B, Chronic"[Mesh] OR

"hepatitis b"[tiab] OR HBV[tiab] OR "hepatitis b virus"[tiab] )

## **2. Web of Science**

TS=("climate change" OR "global warming" OR "greenhouse effect" OR

heatwave* OR "heat wave*" OR heat* OR "hot temperature" OR thermal OR

drought* OR flood* OR wildfire* OR "extreme weather" OR "extreme rainfall")

AND

TS=(pregnancy OR pregnant OR maternal OR antenatal OR prenatal OR

intrapartum OR peripartum OR postpartum OR obstetric* OR mother* OR

neonatal OR neonate* OR newborn* OR perinatal OR infant*)

AND

TS=(HIV OR "human immunodeficiency virus" OR AIDS OR

"acquired immunodeficiency syndrome" OR PLHIV OR

"mother-to-child transmission" OR MTCT OR "vertical transmission" OR

"perinatal transmission" OR "sexually transmitted infection" OR

"sexually transmitted disease" OR STI OR STIs OR STD OR STDs OR

"venereal disease" OR syphilis OR gonorrh*ea OR chlamydia OR

trichomonas* OR "herpes simplex" OR HSV OR "human papillomavirus" OR

HPV OR "Mycoplasma genitalium" OR "Pelvic Inflammatory Disease" OR PID OR

toxoplasmosis OR "Toxoplasma gondii" OR rubella OR cytomegalovirus OR

CMV OR varicella OR chickenpox OR "Varicella-Zoster Virus" OR

parvovirus OR "Parvovirus B19" OR zika OR "group B streptococcus" OR

GBS OR "hepatitis b" OR HBV OR "hepatitis b virus")

## **3. Scopus**

***Set 1: Climate / environment terms***

TITLE-ABS-KEY("climate change" OR "global warming" OR "greenhouse effect" OR

heatwave* OR "heat wave*" OR heat* OR "hot temperature" OR thermal OR

drought* OR flood* OR wildfire* OR "extreme weather" OR "extreme rainfall")

***Set 2: Maternal and neonatal population***

TITLE-ABS-KEY(pregnancy OR pregnant OR maternal OR antenatal OR prenatal OR

intrapartum OR peripartum OR postpartum OR obstetric* OR mother* OR

neonatal OR neonate* OR newborn* OR perinatal OR infant*)

***Set 3: Vertically transmissible infections***

TITLE-ABS-KEY(HIV OR "human immunodeficiency virus" OR AIDS OR

"acquired immunodeficiency syndrome" OR PLHIV OR

"mother-to-child transmission" OR MTCT OR "vertical transmission" OR

"perinatal transmission" OR "sexually transmitted infection" OR

"sexually transmitted disease" OR STI OR STIs OR STD OR STDs OR

"venereal disease" OR syphilis OR gonorrh*ea OR chlamydia OR

trichomonas* OR "herpes simplex" OR HSV OR "human papillomavirus" OR

HPV OR "Mycoplasma genitalium" OR "Pelvic Inflammatory Disease" OR PID OR

toxoplasmosis OR "Toxoplasma gondii" OR rubella OR cytomegalovirus OR

CMV OR varicella OR chickenpox OR "Varicella-Zoster Virus" OR

parvovirus OR "Parvovirus B19" OR zika OR "group B streptococcus" OR

GBS OR "hepatitis b" OR HBV OR "hepatitis b virus")

**Final Combined Search**

#1 AND #2 AND #3
